# Risk for total knee arthroplasty after tibial plateau fractures: a systematic review

**DOI:** 10.1007/s00167-023-07585-8

**Published:** 2023-10-04

**Authors:** D. J. Haslhofer, N. Kraml, P. W. Winkler, T. Gotterbarm, A. Klasan

**Affiliations:** 1 Department for Orthopedics and Traumatology, Med Campus III, Kepler University Hospital Linz, Krankenhausstrasse 9, 4020 Linz, Austria; 2 Faculty of Medicine, Johannes Kepler University Linz, Altenbergerstrasse 69, 4040 Linz, Austria; 3 Department for Orthopedics and Traumatology, AUVA Graz, Göstinger Straße 24, 8020 Graz, Austria

**Keywords:** Tibial plateau fracture, Proximal tibia fracture, TKA, Total knee arthroplasty, Total knee replacement

## Abstract

**Purpose:**

Tibial plateau fractures (TPFs) may lead to posttraumatic osteoarthritis and increase the risk for total knee arthroplasty (TKA). The aim of this systematic review was to analyse the conversion rate to TKA after TPF treatment.

**Methods:**

A systematic search for studies reviewing the conversion rate to TKA after TPF treatment was conducted. The studies were screened and assessed by two independent observers. The conversion rate was analysed overall and for selected subgroups, including different follow-up times, treatment methods, and study sizes.

**Results:**

A total of forty-two eligible studies including 52,577 patients were included in this systematic review. The overall conversion rate of treated TPF to TKA in all studies was 5.1%. Thirty-eight of the forty-two included studies indicated a conversion rate under 10%. Four studies reported a higher percentage, namely, 10.8%, 10.9%, 15.5%, and 21.9%. Risk factors for TKA following TPF treatment were female sex, age, and low surgeon and hospital volume. The conversion rate to TKA is particularly high in the first 5 years after fracture.

**Conclusion:**

Based on the studies, it can be assumed that the conversion rate to TKA is approximately 5%. The risk for TKA is manageable in clinical practice.

**Prospero registration number:**

CRD42023385311.

**Level of evidence:**

IV.

## Introduction

Tibial plateau fractures (TPFs) are injuries of the proximal tibia that can be intraarticular or extraarticular. Treatment of these injuries can be nonoperative or operative. Surgical possibilities are a fixation procedure or primary total knee arthroplasty (TKA). The gold standard fixation method is open reduction and internal fixation (ORIF). Surgical treatment may lead to infection, knee stiffness, non-union, fixation failure, and posttraumatic osteoarthritis (PTOA) [43]. PTOA has been reported to occur relatively frequently in patients with TPF [27, 36]. The latter can lead to knee arthroplasty at an early or later stage. Compared to TKA due to primary osteoarthritis, knee arthroplasty with a previous TPF can present a major challenge due to stiffness, compromised bone quality, and bone stock as well as infection [6, 43].

In elderly patients, ORIF is a challenge due to poor bone quality, metaphyseal bone comminution, and a friable soft tissue envelope. One approach is to treat older patients with knee arthroplasty instead of a fixation procedure, which primarily poses different challenges [22, 52]. The risk of TKA after surgically treated TPF remains unclear.

The objective of this systematic review was to quantify the conversion rate to TKA after TPF treatment. It was hypothesised that the conversion rate is not as high as often assumed.

## Methods

This systematic review was conducted according to Preferred Reporting Items for Systematic Reviews and Meta-Analyses (PRISMA) 2020 Guidelines [33].

### Search strategy

The purpose of this review was to assess the risk of TKA following TPF treatment. A comprehensive systematic search using the PubMed, Medline, and Scopus databases was performed. The search terms were *(“tibial plateau fracture”) OR (“proximal tibia fracture”) AND (“tka”) OR (“total knee arthroplasty”) OR (“total knee replacement”)*. The capitalised words represent Boolean operators. All articles published before December 2022, when the systematic search was conducted, were considered.

### Inclusion criteria

Inclusion criteria comprised a mean follow-up of 1 year and a cohort of at least ten patients. The search was limited to the English and German languages. No study was excluded due to the year of publication. Systematic reviews, conference abstracts, review articles, and expert opinions were not included. Studies reporting on surgically treated TPFs as well as studies reporting on nonoperatively treated TPFs were considered.

### Exclusion criteria

Literature reviewing TPFs treated with primary TKA was excluded. Studies analysing TPF treated with osteochondral allograft, alone or combined with a femoral osteotomy, were also not considered. Studies were not included if the full text was not available. Articles reporting pathological TPF were not examined in this systematic review. Different studies with the same number of patients and TKAs, same follow-up, same authors, and same hospital were considered only once.

### Selection process and data collection

Two authors (Initials) independently reviewed the titles and abstracts of the retrieved articles. Next, duplicates were removed, and full texts were analysed by applying the inclusion and exclusion criteria. In case of disagreement on inclusion, a consensus was reached by discussion with a third author (AK). The references of the retrieved articles were manually screened. Afterwards, the studies were searched for the following data: number of patients with a TPF, follow-up time (mean, minimal, maximal), patients who have undergone TKA, and percent value (TKA/TPF). Missing information (e.g. percent value: TKA/TPF) was calculated manually with the specified data.

The total conversion rate of all studies was calculated as the sum of all patients and all TKAs. Furthermore, the conversion rate was determined for some selected subgroups. The results are presented graphically. The data evaluation was performed by two authors independently. A meta-analysis could not be performed due to the heterogeneity of the study data. Therefore, the content of the studies was analysed through a descriptive procedure.

To assess the trustworthiness, relevance, and results of the published papers, the Joanna Briggs Institute (JBI) critical appraisal checklist for case series [30] was used. Two authors applied the JBI tool independently for assessment of the selected articles. Any disagreement was resolved through discussion with a third author.

## Results

### Search results

**Fig. 1:**
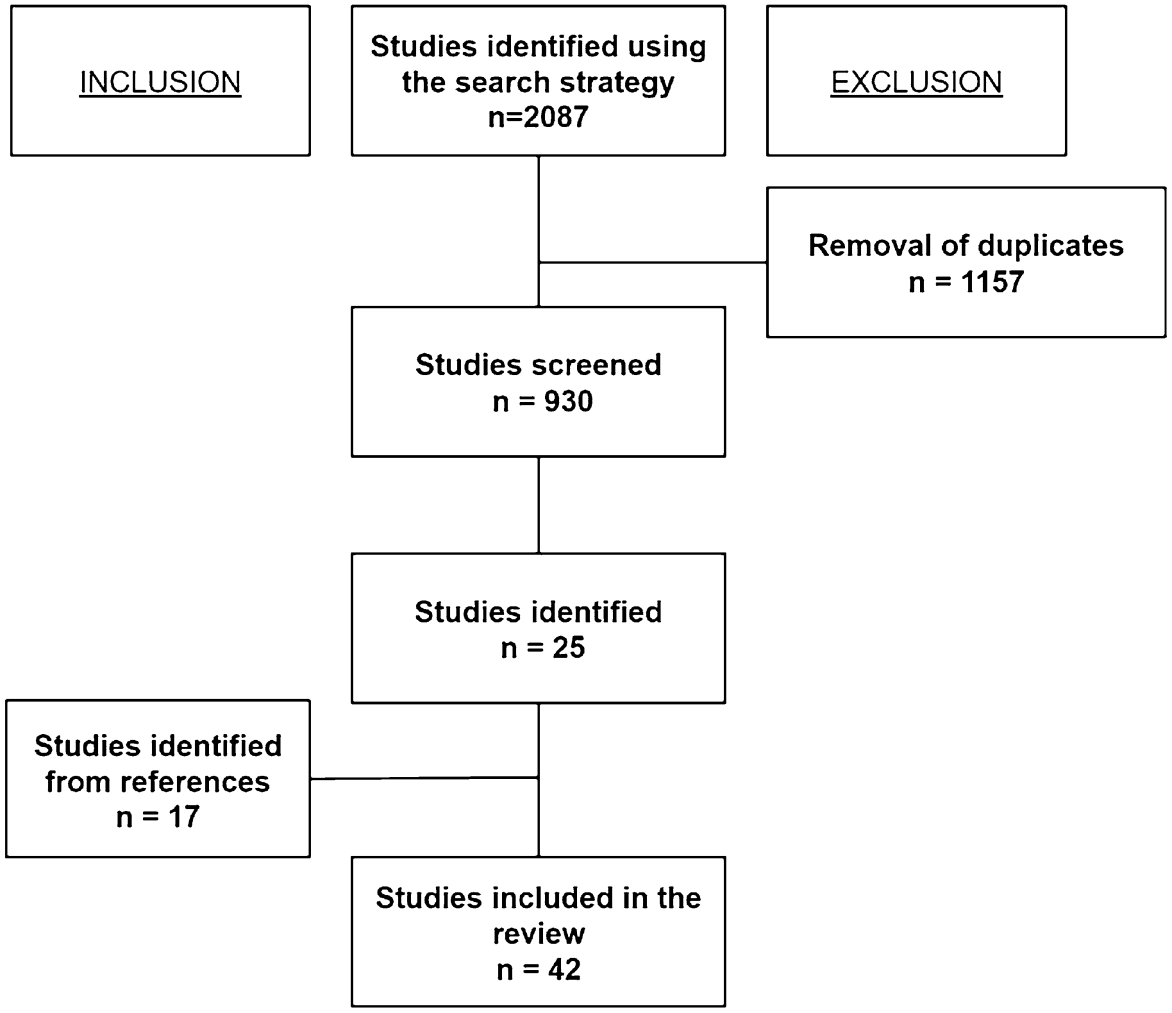
Flowchart showing the literature selection process

A total of 2087 articles were identified through a comprehensive search of the PubMed, Medline, and Scopus databases, resulting in twenty-five studies meeting the inclusion criteria. Through a manual search of the references in the retrieved papers, 17 articles were added. Finally, 42 studies were included in this systematic review (Fig. 1).

### Study characteristics

In total, 52,577 patients were examined in the forty-two studies. The study with the largest number of participants reviewed 22,988 patients [49]. The key characteristics of the identified studies are summarised in Tables 1 and 2.

**Table 1:** List of all included studies: author, year of publication, study type, follow-up time (mean, minimal, maximal) age, male/female

Number	Author	Year of publication	Study type	Follow-up time [years]			Age [years]	Male/female
				Mean	Minimal	Maximal		
1	Kim et al. [25]	2022	Retrospective	2	1	8	56.5	90/63
2	Ochen et al. [31]	2020	Retrospective	7.1	1	NA	53	107/107
3	Pinter et al. [34]	2020	Retrospective	NA	NA	NA	NA	NA
4	Oladeji et al. [32]	2019	Retrospective	1.8	0.5	11	47	192/158
5	Scott et al. [40]	2020	Retrospective	5	NA	NA	64.5	469/808
6	Ali et al. [2]	2003	Prospective	3.1	1.5	4.2	72	NA
7	Wasserstein et al. [51]	2014	Retrospective	10	NA	NA	48.9	4343/4083
8	Elsoe et al. [12]	2019	Retrospective	13.9	NA	NA	52.6	3466/4484
9	Gonzalez et al. [18]	2020	Prospective	5.6	5	10.7	47.9	49/53
10	Tarng et al. [46]	2019	Retrospective	2.8	2	4	53	5/11
11	Hansen et al. [19]	2022	NA	3	NA	NA	NA	NA
12	Warner et al. [50]	2018	Retrospective	2.5	1	9.2	54	40/42
13	Shimizu et al. [41]	2016	Retrospective	4	1	13	72	NA
14	Row et al. [39]	2018	Retrospective	1.3	1	2.8	43	NA
15	Hsu et al. [21]	2001	Retrospective	4.1	3	5.6	66.3	17/3
16	Timmers et al. [47]	2014	Retrospective	6.8	2.6	13.3	64.3	26/56
17	Chan et al. [9]	2012	NA	NA	2	NA	49	NA
18	Kalmet et al. [23]	2019	Retrospective	NA	1	NA	50.8	52/39
19	Manidakis et al. [27]	2010	Retrospective	1.6	1	5.8	52	73/52
20	Su et al. [44]	2004	Retrospective	2.5	NA	NA	66.7	14/24
21	Frattini et al. [17]	2009	Retrospective	4.2	2	11	72	23/26
22	Biyani et al. [4]	1995	Retrospective	3.7	1	7	71.7	NA
23	Ramos et al. [37]	2013	Prospective	NA	1	NA	51	18/12
24	Citak et al. [10]	2019	Retrospective	2.1	1.1	4.6	51.2	15/5
25	Vestergaard et al. [49]	2020	NA	NA	NA	NA	NA	NA
26	Cavallero et al. [8]	2018	Retrospective	2	1	3.4	50	34/22
27	Simpson et al. [42]	2004	Retrospective	NA	1	NA	52.2	12/14
28	Mehin et al. [28]	2012	Retrospective	11	2	17	45.8	NA
29	van Dreumel et al. [48]	2015	Retrospective	6.1	2.9	9.8	NA	NA
30	Roerdink et al. [38]	2001	Retrospective	3	2	5	72	3/27
31	Elsoe et al. [14]	2016	Retrospective	5.2	NA	NA	45.1	17/20
32	Elsoe et al. [13]	2018	Prospective	1	NA	NA	NA	NA
33	Elsoe et al. [15]	2016	Retrospective	2.5	NA	NA	53.8	12/16
34	Krupp et al. [26]	2009	Retrospective	1.1	0.5	4.4	47	23/35
35	Honkonen [20]	1994	NA	7.6	3.3	13.4	50.5	52/78
36	Ali et al. [1]	2003	Prospective	2.5	1.5	4.2	57	8/12
37	Tapper et al. [45]	2022	Retrospective	5.1	NA	NA	57	3269/4432
38	Assink et al. [3]	2022	NA	6.7	NA	NA	53.1	155/379
39	Keightley et al. [24]	2015	Retrospective	7.8	1	19	49	62/43
40	Boldin et al. [5]	2006	Prospective	NA	3	3.4	NA	NA
41	Dall’oca et al. [11]	2012	Retrospective	6.1	1	9.6	51	54/46
42	Rademakers et al. [36]	2007	Retrospective	1	NA	NA	46	112/90

NA, not available

**Table 2:** List of all included studies: author, patients with a TPF, patients who underwent TKA, percent value (TKA/TPF), type of fracture, time to TKA, therapy

Number	Author	Patients	TKA	%	Type of fracture	Time to TKA	Treatment
1	Kim et al. [25]	153	5	3.2	TPF 41B/C	2.8	Operative
2	Ochen et al. [31]	214	6	2.8	Bicondylar TPF	1.9	Operative
3	Pinter et al. [34]	891	19	2.1	TPF	1.7	Operative
4	Oladeji et al. [32]	350	21	6.0	TPF 41B/C	3.7	Operative
5	Scott et al. [40]	1277	58	4.5	TPF	1.5	Operative
6	Ali et al. [2]	11	1	9.0	Bicondylar TPF	NA	Operative
7	Wasserstein et al. [51]	8426	615	7.3	TPF	NA	Operative
8	Elsoe et al. [12]	7950	452	5.7	TPF	NA	Operative and nonoperative
9	Gonzalez et al. [18]	102	1	0.9	Displaced TPF	4	Operative
10	Tarng et al. [46]	16	0	0	3 Column TPF	–	Operative
11	Hansen et al. [19]	56	5	8.9	lateral TPF	1.6	Operative
12	Warner et al. [50]	82	1	1.2	TPF	3	Operative
13	Shimizu et al. [41]	31	0	0	Displaced TPF	–	Operative
14	Row et al. [39]	28	1	3.5	Bicondylar TPF	NA	Operative
15	Hsu et al. [21]	20	0	0	Displaced TPF	–	Operative
16	Timmers et al. [47]	82	18	21.9	TPF	NA	Operative
17	Chan et al. [9]	58	9	15.5	Bicondylar TPF	NA	Operative
18	Kalmet et al. [23]	91	10	10.9	TPF	NA	Operative
19	Manidakis et al. [27]	125	5	4.0	TPF	2.6	Operative and nonoperative
20	Su et al. [44]	38	3	7.9	Displaced TPF	1.1	Operative
21	Frattini et al. [17]	49	2	4.0	TPF	4	Operative
22	Biyani et al. [4]	32	0	0	Displaced TPF	–	Operative
23	Ramos et al. [37]	30	2	6.6	displaced TPF	1.7	Operative
24	Citak et al. [10]	20	1	5.0	Bicondylar TPF	1.2	Operative
25	Vestergaard et al. [49]	22,988	1013	4.4	TPF	NA	Operative and nonoperative
26	Cavallero et al. [8]	56	2	3.5	Bicondylar TPF	NA	Operative
27	Simpson et al. [42]	26	2	7.6	Lateral TPF	1.1	Operative
28	Mehin et al. [28]	286	8	2.7	TPF	NA	Operative and nonoperative
29	van Dreumel et al. [48]	96	7	7.3	TPF	1	Operative
30	Roerdink et al. [38]	30	1	3.3	TPF	2	Operative
31	Elsoe et al. [14]	37	2	5.4	Lateral TPF	NA	Operative
32	Elsoe et al. [13]	24	1	4.1	TPF	NA	Operative
33	Elsoe et al. [15]	28	1	3.5	Lateral TPF	NA	Operative
34	Krupp et al. [26]	58	2	3.4	Bicondylar TPF	NA	Operative
35	Honkonen [20]	130	3	2.3	TPF	NA	Operative and nonoperative
36	Ali et al. [1]	20	1	5.0	Bicondylar TPF	NA	Operative
37	Tapper et al. [45]	7701	340	4.4	TPF	2.1	Operative and nonoperative
38	Assink et al. [3]	534	58	10.8	TPF	NA	Operative and nonoperative
39	Keightley et al. [24]	105	2	1.9	Schatzker IV, V, VI	6.5	Operative
40	Boldin et al. [5]	24	2	8.3	TPF	2.1	Operative
41	Dall'oca et al. [11]	100	2	2.0	TPF	NA	Operative
42	Rademakers et al. [36]	202	2	0.9	TPF	NA	Operative

NA, not available

**Fig. 2:**
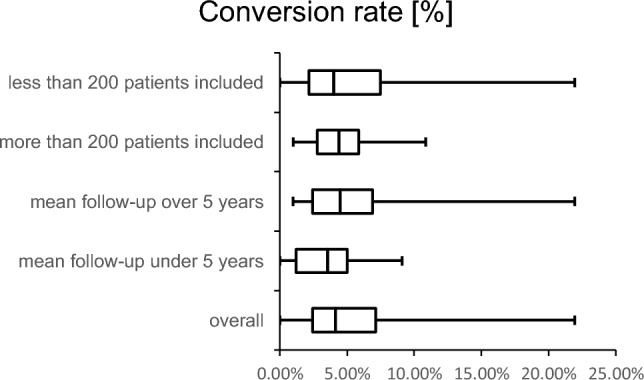
Boxplots showing the conversion rates [%] to total knee arthroplasty: overall, follow-up under 5 years, follow-up over 5 years, studies with more than 200 patients, studies with less than 200 patients (studies comparing treatment methods are not presented due to the small number of studies with nonoperative and operative treated patients)

Thirty-five papers verified outcome after surgical treatment. Seven studies included surgically and non-surgically treated patients [3, 12, 20, 27, 28, 45, 49]. Four studies involved no TKA implantation during the follow-up time. Half of all studies reported a percentage between 2.5% and 7% (Fig. 2). Four publications had a percent value over 10%. The highest conversion rate was reported at 21.9% after a mean follow-up time of 6.8 years.

The overall conversion rate of all patients and TKAs of the included studies was 5.1% (Table 3). Studies with a mean follow-up under 5 years reported that 4.2% of patients underwent TKA, compared to 5.9% in studies with a mean follow-up of more than 5 years. A lower conversion rate to TKA was determined in studies including surgically and non-surgically treated patients (4.6%). When considering only studies with surgically treated patients, the percentage was 6.3%.

**Table 3:** Conversion rate overall and in selected subgroups

Group	Patients	TKA	%
Overall	52,577	2684	5.1
Mean follow-up under 5 years	2706	113	4.2
Mean follow-up over 5 years	25,763	1514	5.9
Operative	12,863	805	6.3
Operative and nonoperative	39,714	1821	4.6
More than 200 patients Included	50,819	2592	5.1
Less than 200 patients included	1758	92	5.2

Table 4 shows the results of the evaluation of the studies using the JBI tool.

**Table 4:** The results of the JBI checklist for case series

		1	2	3	4	5	6	7	8	9	10
1	Kim et al. [25]	Y	Y	Y	U	U	Y	Y	Y	Y	Y
2	Ochen et al. [31]	Y	Y	Y	Y	Y	Y	Y	Y	Y	Y
3	Pinter et al. [34]	Y	Y	Y	U	U	Y	Y	Y	Y	Y
4	Oladeji et al. [32]	Y	Y	Y	Y	Y	Y	Y	Y	Y	Y
5	Scott et al. [40]	Y	Y	Y	Y	Y	Y	Y	Y	Y	Y
6	Ali et al. [2]	Y	Y	Y	Y	Y	Y	Y	Y	Y	Y
7	Wasserstein et al. [51]	Y	Y	Y	Y	Y	Y	Y	Y	Y	Y
8	Elsoe et al. [12]	Y	Y	Y	Y	Y	Y	N	Y	Y	Y
9	Gonzalez et al. [18]	Y	Y	Y	Y	Y	Y	Y	Y	Y	Y
10	Tarng et al. [46]	Y	Y	Y	Y	Y	Y	N	Y	Y	NA
11	Hansen et al. [19]	Y	Y	Y	Y	Y	Y	Y	Y	Y	NA
12	Warner et al. [50]	Y	Y	Y	U	U	Y	Y	Y	Y	Y
13	Shimizu et al. [41]	Y	Y	Y	U	U	Y	Y	Y	Y	Y
14	Row et al. [39]	Y	Y	Y	Y	Y	Y	Y	Y	Y	NA
15	Hsu et al. [21]	Y	Y	Y	U	U	Y	Y	Y	Y	NA
16	Timmers et al. [47]	Y	Y	Y	Y	Y	Y	N	Y	Y	Y
17	Chan et al. [9]	Y	Y	Y	Y	Y	Y	N	Y	Y	Y
18	Kalmet et al. [23]	Y	Y	Y	U	U	Y	N	Y	Y	Y
19	Manidakis et al. [27]	Y	Y	Y	Y	Y	Y	Y	Y	Y	Y
20	Su et al. [44]	Y	Y	Y	Y	Y	Y	Y	Y	Y	Y
21	Frattini et al. [17]	Y	Y	Y	Y	Y	Y	Y	Y	Y	Y
22	Biyani et al. [4]	Y	Y	Y	U	U	Y	Y	Y	Y	Y
23	Ramos et al. [37]	Y	Y	Y	N	N	Y	Y	Y	Y	Y
24	Citak et al. [10]	Y	Y	Y	U	U	Y	Y	Y	Y	Y
25	Vestergaard et al. [49]	Y	Y	Y	Y	Y	Y	Y	Y	Y	Y
26	Cavallero et al. [8]	Y	Y	Y	Y	Y	Y	Y	Y	Y	Y
27	Simpson et al. [42]	Y	Y	Y	NA	NA	Y	N	Y	Y	Y
28	Mehin et al. [28]	Y	Y	Y	Y	Y	Y	Y	Y	Y	Y
29	van Dreumel et al. [48]	Y	Y	Y	Y	Y	Y	Y	Y	Y	Y
30	Roerdink et al. [38]	Y	Y	Y	Y	Y	Y	Y	Y	Y	Y
31	Elsoe et al. [14]	Y	Y	Y	Y	Y	Y	Y	Y	Y	Y
32	Elsoe et al. [13]	Y	Y	Y	Y	Y	Y	Y	Y	Y	Y
33	Elsoe et al. [15]	Y	Y	Y	Y	Y	Y	N	Y	Y	Y
34	Krupp et al. [26]	Y	Y	Y	Y	Y	Y	Y	Y	Y	Y
35	Honkonen [20]	N	Y	Y	U	U	Y	Y	Y	Y	Y
36	Ali et al. [1]	Y	Y	Y	Y	Y	N	Y	Y	Y	Y
37	Tapper et al. [45]	Y	Y	Y	Y	Y	Y	N	Y	Y	Y
38	Assink et al. [3]	Y	Y	Y	Y	Y	Y	Y	Y	Y	Y
39	Keightley et al. [24]	Y	Y	Y	Y	Y	Y	N	Y	Y	Y
40	Boldin et al. [5]	Y	Y	Y	Y	Y	y	Y	Y	Y	Y
41	Dall'oca et al. [11]	Y	Y	Y	U	U	Y	Y	Y	Y	Y
42	Rademakers et al. [36]	Y	Y	Y	Y	Y	Y	N	Y	Y	Y

Question 1: clear criteria for inclusion? 2: condition measured in a standard reliable way? 3: valid methods used for identification of the condition? 4: consecutive inclusion? 5: complete inclusion of all participants? 6: reporting demographics (time period and male/female, age or something else)? 7: reporting of clinical information (at least 3 clinical information: type of fracture, mechanism of injury, comorbidities, or something else)? 8: outcomes or follow-up results clearly reported? 9: reporting demographic information? 10: statistical analysis appropriate?

Y, yes; N, no; U, unclear; NA, not applicable

## Discussion

The most important finding of this systematic review was that the conversion rate to TKA is not as high as often assumed. Thirty-eight of the forty-two articles identified a conversion rate between 0 and 10%. Two studies specified that the percentage value was slightly higher at 10.8% and 10.9% [3, 23]. The highest conversion rates to TKA were 15.5% and 21.9% [9, 47]. The high percentage cannot be deduced from the available study results. Four articles reported no TKA during the follow-up time. This might be a result of the low number of patients in these studies or shorter follow-up.

The sum of all patients with TPF and all TKAs results in a conversion rate of 5.1%. Half of all included studies reported a percentage between 2.5 and 7%. The average risk was approximately 5% when considering only the five largest studies [12, 40, 45, 49, 51]. Based on the available publications, the risk for knee replacement can be expected to be approximately 5%.

Studies that included only surgically treated patients showed a higher conversion rate than studies including non-surgically treated patients. The reason for this might be that patients treated non-surgically had no or minimal displacement, which might influence the onset of PTOA.

ORIF is the gold standard for tibial plateau fracture [35]. Chan et al. [9] compared patients with external fixation to patients with internal fixation after TPF. The study reported no significant difference in outcome between these fixation methods. Some articles investigated the outcome of TPF with other treatment methods, such as the Allring fixator [2], Ilizarov fixator [37], or LISS (less invasive stabilisation system) plate [5]. Due to the small number of participants in the studies with different fixation methods, it was not possible to compare them directly with each other.

Three studies were designed as a matched cohort study. Risk for TKA was compared between patients with a previous TPF and patients without a previous TPF. The publication of Elsoe et al. [12] reported that 5.7% of patients with a previous TPF and 2% of patients without a TPF underwent TKA during the follow-up period. The risk for TKA was 3.5 times higher with a prior TPF. Wasserstein et al. [51] even described a 5.3-fold increased risk for TKA compared with a matched group from the general population. Tapper et al. [45] reported an increase in the risk of 3.2% in the surgically treated group of patients and 1.8% in the nonoperatively treated group compared with a reference group.

The risk for a TKA is particularly high in the first 5 years after the injury. Data from long-term studies show that comparatively fewer prostheses are fitted after 5 years [12, 51]. Scott et al. [40] and Ochen et al. [31] reported a time to TKA as 1.5 years and 1.9 years, respectively. The mean follow-up times in these studies were 7.1 years and 5 years. In relation to the follow-up time, a change to a TKA became necessary at an early stage. By comparing articles with a follow-up of less than 5 years and more than 5 years, we found slightly increased percentages for the 1st and 3rd quartiles and the median with a follow-up of more than 5 years. In summary, it can be assumed that the highest risk of converting to TKA is in the first 5 years after the fracture.

Some publications reported risk factors that led to the implantation of prostheses. The study by Wasserstein et al. [51] pointed out that bicondylar fractures are associated with increased risk of TKA. Eight publications included only bicondylar fractures. However, no increased conversion to TKA was found. In addition, Scott et al. [40] reported a higher conversion rate of unicondylar fractures compared to bicondylar fractures (not statistically significant).

Six publications pointed out a higher conversion rate among women [12, 19, 32, 40, 45, 51]. In the study by Su et al. [44], three patients underwent TKA, and all of them were women. Among other aspects, associations for higher conversion rates with the following were reported: tobacco [32], high BMI [19, 40], comorbidities [51], and a high rate of soft tissue injuries [19]. However, these correlations are not entirely clear. For example, according to Scott et al. [40], tobacco and diabetes are not significant risk factors. Wasserstein et al. [51] found no association between TKA and rural address, open fracture, associated tibial shaft fracture, hospital status, surgeon volume, or after-hours surgery. Brodke et al. [7] explicitly compared the conversion rate between hospitals and surgeons with high and low caseloads. It was detected that high-volume treatment of TPF reduced the risk of TKA. It can be assumed that low case numbers and female sex are risk factors for TKA after a TPF.

The included studies referred to controversial statements about age as a risk factor for TKA. The study by Kim et al. [25] compared the need for TKA between elderly and younger patients. No significant difference was found between these two age groups. Seven studies in this systematic review included only elderly patients [2, 4, 17, 21, 38, 41, 44]. No increased conversion to TKA was observed. However, the number of study participants and the follow-up time were significantly low. Three large studies [12, 45, 51] reported increased risk in elderly patients for TKA after TPF. Furthermore, these publications had a long follow-up time after the fracture. Additionally, the literature states that older age probably leads to poorer fracture healing [16, 29]. Therefore, it can be assumed that older age leads to more TKAs after TPF.

This systematic review has some limitations. Only five studies with more than a thousand patients were included. Most publications reviewed fewer than 100 study participants. In some studies, the mean follow-up time was short. Different treatment methods were used in the studies. Therefore, articles with different study sizes, follow-up, and treatment methods were compared with each other in terms of conversion to TKA, risk factors, and time to TKA.

Implications of this review: The risk of TKA following TPF is not as high as often assumed. Non-operative therapy and joint preservation surgery are good treatment methods, and the risk for TKA is manageable. Based on the publications, a conversion rate of approximately 5% can be concluded. The risk for TKA after TPF is manageable in clinical practice.

## Conclusion

The conversion rate ranged from 0 to 21%. Based on the available data, risk for TKA can be assumed to be approximately 5%. Risk factors for TKA are female sex, elderly, and low surgeon and hospital volume. The risk for TKA increases in the first 5 years following TPF.

## Abbreviations

TPF: Tibial plateau fracture
TKA: Total knee arthroplasty
ORIF: Open reduction and internal fixation
PTOA: Posttraumatic osteoarthritis

## References

[1] Ali AM, Burton M, Hashmi M, Saleh M (2003). Outcome of complex fractures of the tibial plateau treated with a beam-loading ring fixation system. J Bone Jt Surg Br.

[2] Ali AM, Burton M, Hashmi M, Saleh M (2003). Treatment of displaced bicondylar tibial plateau fractures (OTA-41C2&3) in patients older than 60 years of age. J Orthop Trauma.

[3] Assink N, Kraeima J, Meesters AML, El Moumni M, Bosma E, Nijveldt RJ (2022). 3D assessment of initial fracture displacement of tibial plateau fractures is predictive for risk on conversion to total knee arthroplasty at long-term follow-up. Eur J Trauma Emerg Surg.

[4] Biyani A, Reddy NS, Chaudhury J, Simison AJ, Klenerman L (1995). The results of surgical management of displaced tibial plateau fractures in the elderly. Injury.

[5] Boldin C, Fankhauser F, Hofer HP, Szyszkowitz R (2006). Three-year results of proximal tibia fractures treated with the LISS. Clin Orthop Relat Res.

[6] Brockman BS, Maupin JJ, Thompson SF, Hollabaugh KM, Thakral R (2020). Complication rates in total knee arthroplasty performed for osteoarthritis and post-traumatic arthritis: a comparison study. J Arthroplasty.

[7] Brodke DJ, Morshed S (2021). Low surgeon and hospital volume increase risk of early conversion to total knee arthroplasty after tibial plateau fixation. J Am Acad Orthop Surg.

[8] Cavallero M, Rosales R, Caballero J, Virkus WW, Kempton LB, Gaski GE (2018). Locking plate fixation in a series of bicondylar tibial plateau fractures raises treatment costs without clinical benefit. J Orthop Trauma.

[9] Chan C, Keating J (2012). Comparison of outcomes of operatively treated bicondylar tibial plateau fractures by external fixation and internal fixation. Malays Orthop J.

[10] Citak C, Kayali C, Ozan F, Altay T, Karahan HG, Yamak K (2019). Lateral locked plating or dual plating: a comparison of two methods in simple bicondylar tibial plateau fractures. Clin Orthop Surg.

[11] Dall'oca C, Maluta T, Lavini F, Bondi M, Micheloni GM, Bartolozzi P (2012). Tibial plateau fractures: compared outcomes between ARIF and ORIF. Strateg Trauma Limb Reconstr.

[12] Elsoe R, Johansen MB, Larsen P (2019). Tibial plateau fractures are associated with a long-lasting increased risk of total knee arthroplasty a matched cohort study of 7950 tibial plateau fractures. Osteoarthr Cartil.

[13] Elsoe R, Larsen P, Petruskevicius J, Kold S (2018). Complex tibial fractures are associated with lower social classes and predict early exit from employment and worse patient-reported QOL: a prospective observational study of 46 complex tibial fractures treated with a ring fixator. Strateg Trauma Limb Reconstr.

[14] Elsoe R, Larsen P, Shekhrajka N, Ferreira L, Ostgaard SE, Rasmussen S (2016). The outcome after lateral tibial plateau fracture treated with percutaneus screw fixation show a tendency towards worse functional outcome compared with a reference population. Eur J Trauma Emerg Surg.

[15] Elsøe R, Larsen P, Rasmussen S, Hansen HA, Eriksen CB (2016). High degree of patient satisfaction after percutaneous treatment of lateral tibia plateau fractures. Dan Med J.

[16] Foulke BA, Kendal AR, Murray DW, Pandit H (2016). Fracture healing in the elderly: a review. Maturitas.

[17] Frattini M, Vaienti E, Soncini G, Pogliacomi F (2009). Tibial plateau fractures in elderly patients. Chir Organi Mov.

[18] Gonzalez LJ, Hildebrandt K, Carlock K, Konda SR, Egol KA (2020). Patient function continues to improve over the first five years following tibial plateau fracture managed by open reduction and internal fixation. Bone Jt J.

[19] Hansen L, Larsen P, Elsoe R (2022). Characteristics of patients requiring early total knee replacement after surgically treated lateral tibial plateau fractures: a comparative cohort study. Eur J Orthop Surg Traumatol.

[20] Honkonen SE (1994). Indications for surgical treatment of tibial condyle fractures. Clin Orthop Relat Res.

[21] Hsu CJ, Chang WN, Wong CY (2001). Surgical treatment of tibial plateau fracture in elderly patients. Arch Orthop Trauma Surg.

[22] Huang JF, Shen JJ, Chen JJ, Tong PJ (2016). Primary total knee arthroplasty for elderly complex tibial plateau fractures. Acta Orthop Traumatol Turc.

[23] Kalmet PHS, Van Horn YY, Sanduleanu S, Seelen HAM, Brink PRG, Poeze M (2019). Patient-reported quality of life and pain after permissive weight bearing in surgically treated trauma patients with tibial plateau fractures: a retrospective cohort study. Arch Orthop Trauma Surg.

[24] Keightley AJ, Nawaz SZ, Jacob JT, Unnithan A, Elliott DS, Khaleel A (2015). Ilizarov management of Schatzker IV to VI fractures of the tibial plateau: 105 fractures at a mean follow-up of 7.8 years. Bone Jt J.

[25] Kim JK, Hwang KT, Soh HS, Shon OJ, Park KC (2022). Comparison of tibial plateau fracture surgical outcomes between young and elderly patients: are outcomes really poorer in the elderly?. Arch Orthop Trauma Surg.

[26] Krupp RJ, Malkani AL, Roberts CS, Seligson D, Crawford CH, Smith L (2009). Treatment of bicondylar tibia plateau fractures using locked plating versus external fixation. Orthopedics.

[27] Manidakis N, Dosani A, Dimitriou R, Stengel D, Matthews S, Giannoudis P (2010). Tibial plateau fractures: functional outcome and incidence of osteoarthritis in 125 cases. Int Orthop.

[28] Mehin R, O'Brien P, Broekhuyse H, Blachut P, Guy P (2012). Endstage arthritis following tibia plateau fractures: average 10-year follow-up. Can J Surg.

[29] Meinberg EG, Clark D, Miclau KR, Marcucio R, Miclau T (2019). Fracture repair in the elderly: clinical and experimental considerations. Injury.

[30] Munn Z, Barker TH, Moola S, Tufanaru C, Stern C, McArthur A (2020). Methodological quality of case series studies: an introduction to the JBI critical appraisal tool. JBI Evid Synth.

[31] Ochen Y, Peek J, McTague MF, Weaver MJ, van der Velde D, Houwert RM (2020). Long-term outcomes after open reduction and internal fixation of bicondylar tibial plateau fractures. Injury.

[32] Oladeji LO, Dreger TK, Pratte EL, Baumann CA, Stannard JP, Volgas DA (2019). Total knee arthroplasty versus osteochondral allograft: prevalence and risk factors following tibial plateau fractures. J Knee Surg.

[33] Page MJ, Moher D, Bossuyt PM, Boutron I, Hoffmann TC, Mulrow CD (2021). PRISMA 2020 explanation and elaboration: updated guidance and exemplars for reporting systematic reviews. BMJ.

[34] Pinter Z, Jha AJ, McGee A, Paul K, Lee S, Dombrowsky A (2020). Outcomes of knee replacement in patients with posttraumatic arthritis due to previous tibial plateau fracture. Eur J Orthop Surg Traumatol.

[35] Prat-Fabregat S, Camacho-Carrasco P (2016). Treatment strategy for tibial plateau fractures: an update. EFORT Open Rev.

[36] Rademakers MV, Kerkhoffs GM, Sierevelt IN, Raaymakers EL, Marti RK (2007). Operative treatment of 109 tibial plateau fractures: five- to 27-year follow-up results. J Orthop Trauma.

[37] Ramos T, Ekholm C, Eriksson BI, Karlsson J, Nistor L (2013). The Ilizarov external fixator–a useful alternative for the treatment of proximal tibial fractures. A prospective observational study of 30 consecutive patients. BMC Musculoskelet Disord.

[38] Roerdink WH, Oskam J, Vierhout PA (2001). Arthroscopically assisted osteosynthesis of tibial plateau fractures in patients older than 55 years. Arthroscopy.

[39] Row ER, Komatsu DE, Watson JT, Jones C, Kottmeier S (2018). Staged prone/supine fixation of high-energy multicolumnar tibial plateau fractures: a multicenter analysis. J Orthop Trauma.

[40] Scott BL, Lee CS, Strelzow JA (2020). Five-year risk of conversion to total knee arthroplasty after operatively treated periarticular knee fractures in patients over 40 years of age. J Arthroplasty.

[41] Shimizu T, Sawaguchi T, Sakagoshi D, Goshima K, Shigemoto K, Hatsuchi Y (2016). Geriatric tibial plateau fractures: clinical features and surgical outcomes. J Orthop Sci.

[42] Simpson D, Keating JF (2004). Outcome of tibial plateau fractures managed with calcium phosphate cement. Injury.

[43] Softness KA, Murray RS, Evans BG (2017). Total knee arthroplasty and fractures of the tibial plateau. World J Orthop.

[44] Su EP, Westrich GH, Rana AJ, Kapoor K, Helfet DL (2004). Operative treatment of tibial plateau fractures in patients older than 55 years. Clin Orthop Relat Res.

[45] Tapper VS, Pamilo KJ, Haapakoski JJ, Toom A, Paloneva J (2022). Risk of total knee replacement after proximal tibia fracture: a register-based study of 7,841 patients. Acta Orthop.

[46] Tarng YW, Lin KC (2019). A combined prone and supine approaches for complex three column tibial plateau fracture with posterolateral articular injury. Injury.

[47] Timmers TK, van der Ven DJ, de Vries LS, van Olden GD (2014). Functional outcome after tibial plateau fracture osteosynthesis: a mean follow-up of 6 years. Knee.

[48] van Dreumel RL, van Wunnik BP, Janssen L, Simons PC, Janzing HM (2015). Mid- to long-term functional outcome after open reduction and internal fixation of tibial plateau fractures. Injury.

[49] Vestergaard V, Becic Pedersen A, Borbjerg Hare K, MorvilleSchrøder H, Troelsen A (2020). Knee fracture increases TKA risk after initial fracture treatment and throughout life. Clin Orthop Relat Res.

[50] Warner SJ, Garner MR, Schottel PC, Fabricant PD, Thacher RR, Loftus ML (2018). The effect of soft tissue injuries on clinical outcomes after tibial plateau fracture fixation. J Orthop Trauma.

[51] Wasserstein D, Henry P, Paterson JM, Kreder HJ, Jenkinson R (2014). Risk of total knee arthroplasty after operatively treated tibial plateau fracture: a matched-population-based cohort study. J Bone Jt Surg Am.

[52] Wong MT, Bourget-Murray J, Johnston K, Desy NM (2020). Understanding the role of total knee arthroplasty for primary treatment of tibial plateau fracture: a systematic review of the literature. J Orthop Traumatol.

